# Hospital and Institutional News

**Published:** 1916-10-14

**Authors:** 


### HOSPITAL AND INSTITUTIONAL NEWS.

### THE DIAMOND JUBILEE OF THE GREAT NORTHERN CENTRAL HOSPITAL.

On October 18, which is St. Luke's Day and therefore especially appropriate for efforts in aid of any medical charity, a meeting will be held at the Mansion House to mark the diamond jubilee of the Great Northern Central Hospital, Holloway Road, N. The Lord Mayor will preside, and the list of those who have promised their support contains many notable names, including a large proportion of ladies. Peers, bishops, soldiers, statesmen, merchant princes, novelists, journalists, and many other professions are represented in the masculine section of the list; while among the ladies are names as widely and as honourably known as any of those among the men. The nature and scope of the work of this hospital are well enough known in institutional and medical circles; but its situation in a busy centre of industrial life is to some extent detrimental to its finances, because many of the beneficent wealthy never have their attention drawn to its existence. The Mansion House meeting should serve as a counterpoise to this disadvantage, and we trust will also result in a very substantial addition to the funds of the hospital.

### THE MEMORY OF EDITH CAVELL.

The first anniversary of the death of Edith Cavell, which took place on October 12 last year, has been signalised by her sister, Miss F. M. Scott Cavell, by the formulation of her proposal to establish homes of rest for nurses, a project to which Miss Cavell had intended to devote herself after her work in Belgium was accomplished. Her idea was to found homes where nurses in need of temporary mental and physical rest could be received until once more able to resume their work. A Council has already been formed to put this excellent scheme into practice, and the list of those who have already consented to serve on it is ample guarantee that the funds collected will be wisely administered. The Council are satisfied that there is a definite need for such homes, and that during the war and for some time afterwards the need will be especially acute. A freehold house standing in three and a-half acres of ground in a suitable locality has been offered as a gift, on the condition that a fund sufficient for its permanent endowment can be raised; it will provide for the needs of about a hundred nurses yearly. When the Council issues its appeal we trust that not only will sufficient funds be secured to enable this offer to be accepted, but that there will be a surplus available for further expansion of the scheme.

### PROPOSAL TO CHANGE THE NAME OF A HOSPITAL.

The Royal Prince Alfred Hospital at Sydney is one of the better-known hospitals of Australia. Its name was adopted shortly after a visit of one of our sailor Princes—the late Duke of Edinburgh, who afterwards became Duke of Saxe-Coburg and Gotha. This Prince was much better known to a former generation than this, and the board of management, as announced at the annual general meeting, feel that it would be fitting and more in accordance with patriotic feeling if the hospital were renamed after our present beloved King and Queen. This announcement was received with great enthusiasm, and an application has been made to the Minister of Health, who, it appears, is the authority in these matters, that the name may be changed to the Royal George and Mary Hospital. Their Majesties when Prince and Princess of Wales visited the hospital in 1901, and laid the foundation-stone of the Queen Victoria Memorial Pavilion. Admittedly there is much to be said for this particular proposal; but the principle needs caution in its application. No one in England now takes much interest in Queen Charlotte, for example; but we should be very sorry to hear of a certain hospital at Marylebone changing its name.

### THE NEW LEEDS HOSPITAL BLOCK.

With the larger aspects of the extension scheme now in part completed at Leeds General Infirmary we propose to deal in a later issue. Here it is desirable to summarise in brief the chief points in the new hospital block, now practically ready for the hundred patients for which it is able to provide. Three storeys high, with a flat roof, and standing at the back of the Town Hall, each is 120 feet in length, with a verandah at one end and the sanitary block at the base. In the new theatres, not yet completed, ventilation is secured by the open-window, not the Plenum, system, and the device of double windows has been adopted in such a way as to prevent draughts. The new theatres, however, are dependent upon the completion of the new boiler-house, which, like the great corridor, still remains to be finished. It should be added that £150,000 was asked for when the scheme for an extension, which should be a memorial to King Edward VII., was undertaken. Of this sum £120,000 has been subscribed and spent, and the seal will be put upon the undertaking when the time comes for appealing for the remainder, so that the great project now nearing completion may be carried through on the scale that was originally planned.

### PATRIOTISM AT BRADFORD.

Princess Marie Louise of Schleswig-Holstein visited Bradford last week, and on Thursday formally opened the extension to the War Hospital, for which the public of the city, at the call of the Lord Mayor, have provided £50,000 in three or four weeks. The extension consists of three new pavilions close to the hospital, which was previously known as St. Luke's. Two of the blocks are of a temporary type, but the third is intended as a permanent addition. They are each 200 feet long by 42 feet wide, with main annexes measuring 40 feet by 25 feet. Large verandahs on the west side are entered from the inside by French windows. The two temporary pavilions are of timber, with brick foundations, and are weather-boarded on the exterior and match-boarded in the interior. The boarded roofs are felt-covered. The third block is built of brick, with fireproof cavity walls; the roof is constructed of Welsh slates, with red ridge tiling. Steam radiators and electric lighting installations are part of the equipment of each pavilion. The former residents at St. Luke's Hospital have been removed to Bowling Park, and a portion of the £50,000 raised by the Lord Mayor has been expended in providing buildings for their accommodation there. The administrator at the War Hospital is Lieutenant-Colonel Wrangham.

### A MILITARY ORTHOPÆDIC HOSPITAL AT CARDIFF.

What Roehampton is doing so marvellously for soldiers broken in the war Cardiff has made up its mind to emulate. Chiefly through the enthusiastic efforts of Mr. Lynn Thomas, C.B., who has been ably seconded by the Lord Mayor of Cardiff (also a medical man), a scheme for the conversion of admirably adapted premises known as the Mansion House, with a garden and two adjacent houses, into an orthopædic convalescent home has been sanctioned by the War Office and the Admiralty. Already £20,000 has been subscribed for setting up the hospital. The honorary treasurers, Mr. J. P. Cadogan and Mr. Percy Miles, have each given £5,000; Messrs. Morgan, Wakely and Co. £2,500; and "A Friend" has forwarded the remaining £7,500. The premises have been purchased, and an appeal is being made to the public for an additional £1,000 for the purchase of "comforts" for the hospital. It is safe to say the money will be forthcoming practically immediately. A deputation from Cardiff inspected Roehampton last week, and it was subsequently announced that Cardiff would take that hospital as its model.

### AN UNFORTUNATE CONTROVERSY AT LEEDS.

A regrettable controversy, which has arisen at Leeds in connection with the Workpeople's Hospital Fund, has involved the resignation of Mr. F. R. Spark, who founded the Fund nearly thirty years ago and for many years has been its chairman. The unfortunate dispute between the chairman and the executive committee concerns a gift of £1,000 made to the Fund in 1907 by Mr. Archibald Ramsden. Mr. Spark charges the executive with having failed to carry out what he alleges was Mr. Ramsden's wish—viz., that the money should be used for the establishment of a children's home. This is the crux of the matter, though other issues have been introduced. The executive say no such wish was expressed, and that nothing was heard of any such desire until 1914, when Mr. Spark wrote to Mr. Ramsden a letter which was not authorised or brought before the executive. In order to meet Mr. Spark they have offered, while denying that the letter did or could create any trust, to submit it to the opinion of counsel, but Mr. Spark declines to accept such a course, describing is as "amazing and painful." The gift was, as a matter of fact, included in the general income of the Fund, and used in the support of the convalescent homes at Horsforth and Ilkley. A special meeting of the general committee of the Fund was held last week, the Lord Mayor presiding, when a full statement on the affair was made by the executive, in whom a vote of confidence was unanimously passed. Great regret was expressed at the loss of Mr. Spark's splendid services, but the executive said they could not allow the "judgment, wishes, or ruling of one man" to over-rule an executive committee composed of forty representatives elected by the workpeople as trustees and managers of the money contributed by them.

### HOSPITAL'S HALF-MILE OF PENNIES.

In order to augment the funds of the Livingstone College Auxiliary Hospital, Mrs. Carter, an enthusiastic supporter of the institution, set out some time ago with the object of obtaining half a mile of pennies—which number, should anyone of a mathematical turn of mind care to work out, represents about £110—and it is gratifying to learn that the attempt has become an accomplished fact. It is also interesting to know that nearly all the amounts received have been in pennies, subscribed in envelopes containing ten, while a great impetus has been given to the scheme by the children attending the Leyton Council Schools, as well as by local Boy Scouts.

### BIRMINGHAM HOSPITAL SATURDAY FUND DISTRIBUTION.

The distribution list recommended by the Executive Committee of the Birmingham Hospital Saturday Fund was presented to the Board of Delegates for confirmation on October 11. Subject to that body's approval, the institutions named will receive in all £12,000, distributed as follows: the General Hospital, £3,780; Queen's, £2,520; General Dispensary, £1,217 (of which £600 is earmarked for the Relief Fund); Children's Hospital, £960; Eye Hospital, £420; Birmingham and Midland Counties Sanatorium, £100; Homœopathic Hospital, £240; Women's Hospital, £600; Lying-In Charity, £240; Orthopædic Hospital, £360; Ear and Throat Hospital, £150; Dental Hospital, £60; Skin and Urinary Hospital, £240; District Nursing Society, £180; Jaffray Hospital, £480; Moseley Hall Convalescent Home, £240; and smaller sums, mostly of £10 or £12, to fifteen smaller charities, nearly all of them nursing societies. This list shows that the Executive Committee take careful note of the smaller as well as of the larger institutions; and that the Birmingham Saturday Fund is of great importance in the budgets of all the Birmingham hospitals, great and small.

### THE BIRMINGHAM ORTHOPÆDIC HOSPITAL.

The annual meeting of the Royal Orthopædic and Spinal Hospital, Birmingham, was held at the institution, Newhall Street, on October 3. In moving the adoption of the report, the chairman, Mr. Edward Ansell, made the observation that the income of the charity is altogether inadequate to its needs; the hospital's value and usefulness are constantly increasing, and the calls upon its services grow steadily greater. He appealed for the sympathy and support of all classes of the community, an appeal which in a town like Birmingham has only to be prosecuted with vigour and energy to be successful. The chairman of the committee, Mr. G. A. Bryson, showed in the report that the expenditure for the year has increased by £532, whereas the income has expanded but £430. Though the adverse balance on the year's work is thus seen to be only £102, nevertheless the deficit is regularly increasing and is naturally a source of anxiety to the committee. Considering the high cost of food and of much else that a hospital has to purchase, the comparatively small rise in expenditure is not unsatisfactory; apparently this hospital does fairly well out of gifts in kind. It is now approaching a century of existence, and has a good record as well as many friends; but it must endeavour to broaden its basis of support, and a resolute policy to this effect should be steadfastly kept in mind by the management.

### EXTENSION OF THE MILITARY HOSPITAL AT BRIGHOUSE.

The War Office authorities have asked the local committee which manages the V.A.D. hospital at Brighouse to double the present accommodation for wounded soldiers there; and to this request the committee has acceded. For the last year about forty beds have been available at this hospital, in a large mansion known as "Boothroyde"; and an additional twenty beds have been located during the summer in tents pitched in the grounds. Now it has been decided to take over and to equip a neighbouring residence, "Longroyde," which was devoted to the housing of Belgian refugees in 1914. This will provide for an additional forty to fifty patients. The work of preparation for the transformation is in progress, and a good proportion of the money is in hand for defraying the initial cost. This extension and the responsibility for a hundred or so of soldier patients is highly to the credit of Brighouse, whose inhabitants are evidently both practical and patriotic people.

### FIRST AID AT THE ROYAL COLLEGE OF SURGEONS.

The Royal College of Surgeons has just made arrangements for two novel courses of lectures, one designed wholly for first-aid and ambulance students, the other for practitioners as well. The principal course is on the Anatomy of the Human Body, and will be given by Professor Keith, the Conservator of the Museum, on Mondays, Wednesdays, and Fridays in November, beginning on the first of the month. The lectures will be illustrated by anatomical preparations and specimens, and these will not only be on view before the lectures in the afternoons, but also for the whole of the following day. A second series of lectures is projected early in the new year. The other course is one of museum demonstrations, by Messrs. Shattock and Colyer, beginning on October 16. It is of less interest, perhaps, for V.A.D. students; but both these series of popular lectures are evidences of an up-to-date and progressive spirit which has not always characterised the Royal College of Surgeons, and is very much to be commended, especially in times like these.

### A SERIOUS ALLEGATION AGAINST NURSING HOMES.

A correspondent of the *Daily Express*, who signs himself "M.D.," and writes from a Marylebone address, brings a very serious charge against the managers of certain (unspecified) private nursing homes. If the writer of the letter is, as would appear to be suggested, a physician in the Marylebone district, he must be presumed to have opportunities of learning the truth, since in that neighbourhood are congregated the majority of the higher class private nursing homes of London. His charges are therefore disquieting, though in the absence of any direct statement as to the names of delinquents, it is impossible to be certain how far they can be substantiated. Briefly, he states that the proprietors of certain private nursing homes, where military patients are accepted under contract with the War Office, are using the patriotic services of voluntary unpaid helpers in place of labour for which they should be paying, and are thus wrongfully enriching themselves. Thousands of women, he says, are acting as honorary nurses, assistant nurses, cooks, housemaids, and parlourmaids in the belief that they are serving their country and their country's soldiers. In regard to some of the private nursing homes they are, he says, merely assisting the proprietors to make their fortunes. The nursing home is run for profit, and is sufficiently paid by the Government to enable this to be done. If voluntary unpaid labour is utilised in such homes, the proprietors should be compelled to hand over to charity a sum equivalent to the amount of the salaries and wages they would be paying in the ordinary way. If any cases of this kind of abuse are brought to our notice and substantiated, we shall be glad to take the matter up on the lines suggested in "M.D.'s" letter.

### NEW WORK AT WORCESTER INFIRMARY.

A special meeting of the governors of Worcester General Infirmary has decided to alter the rule so as to permit the treatment of venereal diseases at the hospital. The executive committee has recommended the following scheme, which provides for the appointment of a medical officer to deal with in- and out-patients of this class, together with the setting aside of two small wards, with two beds in each, in the isolation block. This is a separate building, and can thus be conveniently used for institutional treatment. The financial side of the scheme takes the form of offering to co-operate with the County and City Councils in the national work, provided that each pays not less than £100 in advance, and that at the end of each quarter an account be rendered of the expenses incurred by the infirmary, including a reasonable salary for the medical officer, and that "any excess over the sum advanced be reimbursed." In view of the fact that the amount of the work cannot at present be foreseen no sum is mentioned, but the governors feel that they must not divert any of the ordinary money subscribed to this work, the total cost of which must be borne by the local authorities. The plan therefore is drafted so as to undertake the new work without interfering with the ordinary activities of the hospital.

### THE FATE OF THE MILITARY MENTAL PATIENT.

At a recent meeting of Govan District Board of Control an interesting memorandum was read on the subject of military patients in mental hospitals by Dr. Macdonald, medical superintendent at Hawkhead. He pointed out that if it were desired to admit men who had become insane through active service as private patients at slightly above the so-called pauper rate, so as to avoid the stigma of pauperism, then it was desirable that only such sailors and soldiers should be admitted as had already received treatment in special war hospitals for mental cases. To send such men direct to district mental hospitals would deprive them of valuable advantages, including that of not being certified as insane. Certification, on the other hand, must precede direct admission into the district mental hospitals. There are thus two stigmata to avoid, the stigma of certification in an asylum, and the stigma of pauperism. But, besides this, it is a positive advantage, Dr. Macdonald declares, for all mental cases among wounded men to receive skilled treatment in special hospitals, after which only those found unlikely to improve need be transferred to the mental hospitals of their own districts. If the number of the latter cases should prove too large for the existing hospitals to receive, special accommodation should be provided. We are glad to note that it was decided to forward this able memorandum to the General Board of Control for Scotland.

### THE MELBOURNE HOSPITAL AND THE STATE.

The recently published report of the committee of management of the Melbourne Hospital shows that the condition of the finances is a matter of grave concern. The excess of expenditure over income in the maintenance account for the year ending June 30 was £13,342 and the accumulated deficit £26,463. While the income of the institution has since 1911-12 increased by only £6,000, the expenditure has risen from £28,278 to £52,056. The report complains that the contribution of the Government, which has remained at about £10,000 for each of the last five years, should be more in accordance with the needs of the institution. The answer of the Government is that any money required should be taken from the endowment fund; this suggestion rather bears out the general impression of the medical profession in Australia that the parsimony of the ruling powers is deliberate, and that the era of complete State control is to be hastened by a process of starvation. Another ground for this suspicion is that when the hospital was housed in the poor, inadequate, original buildings the State grant was £15,000 a year, and that it is only since the present efficient institution has been provided, chiefly by public beneficence, that the subsidy has fallen to the present figure.

### THE LESSON OF CONSUMPTION STATISTICS.

A broad view of the value and results of after-care in the case of tuberculous patients was given by Mr. W. Jones, clerk to the Glasgow Insurance Committee, in his evidence before the Commission of Investigation appointed by the Faculty of Insurance to investigate National Health Insurance. He stated that the Insurance Committee administered sanatorium benefit to insured persons only, and not to their dependants, but that 98 per cent. of the 880 patients whose cases were undertaken in 1915 had received institutional treatment. As a result of his exhaustive inquiry into the after-history of patients discharged from sanatoria, he found that about one-third of the applicants were "early," one-third "intermediate," and one-third "advanced" cases. Of the first group 87 per cent. improved, and of the second and third 58 per cent.; while 70 per cent. of early cases were able on dismissal to continue their employment, though the same was true of 36 per cent. only of the intermediate and advanced cases. In regard to housing, Mr. Jones said that about 60 per cent. of the Glasgow population lived in two rooms. Could the number be reduced to 30 per cent. he thought a great reduction of cases would ensue, although, he added, the incidence of tuberculosis in England was not much less than in Glasgow or Scotland.

### WAGES AND DISEASE: AN ECONOMIC PARADOX.

Mr. W. Jones, in the course of his evidence, further disclosed a remarkable fact concerning the treatment of tuberculosis which deserves to be considered in the light of his remarks about housing given in the preceding paragraph. He stated that the Glasgow Committee could not fill their sanatoria at present, although there were cases in the city. He explained this by remarking that the industrial conditions were such that people preferred to remain at work, with the result that the cases applied for sanatorium treatment later. In other words, rather than lose the opportunity of high wages now to be had, people stuck to their work to the last possible moment. This is not only understandable but highly interesting, for we all know that bad housing, malnutrition, overcrowding, as factors which produce tuberculosis, are only the fruits of poverty, which is the basis of them all. It might be supposed, therefore, that higher wages would be followed by a smaller number of intermediate or advanced cases. Mr. Jones explains that this is not so, but the reason, of course, is not that poverty is not the cause of much consumption, but that prosperity under present conditions is so precarious that natural temptation of higher wages is irresistible when it comes.

### BUTTER VERSUS MARGARINE IN HOSPITALS.

Taking their courage in both hands the Manchester Board of Guardians have decided to effect an economy of £8,000 a year by the simple expedient of substituting margarine for butter in the dietary tariff of the officials and sick inmates. The saving named is, of course, estimated on the present high price of butter. There still seems to remain a strange prejudice against margarine on the part of the staffs of various institutions, and the West Ham nurses have asked the Guardians to replace it by butter, promising to relinquish their cake allowance if this is done.

### TOBACCO AND THE SOLDIER.

AN outspoken letter to the *Times*, by Sir Thomas Fraser, on the abuse of tobacco by soldiers, has raised a storm of angry replies as well as a distinct measure of agreement from some of those who have had opportunities of seeing the results of excessive smoking upon the heart of the soldier, and especially of the young soldier. THE HOSPITAL can claim to have been one of the earliest journals to ventilate this important question in an article published February 19, 1916, p. 449. It was then pointed out, and the correspondence of all sorts which has followed Sir Thomas Fraser's letter in the *Times* has only strengthened that opinion, that distinct and sometimes serious symptoms of nicotine poisoning are much too common among soldiers at the Front. These symptoms have long been familiar to the officers of the R.A.M.C. abroad; they are nearly always displayed in cardiac dilatation and irregularity, and the too-lavish distribution of free tobacco and cigarettes is one of the principal factors in producing a state of things which seriously militates against the military efficiency of our Forces. On the other hand, it is not desirable that soldiers should be deprived altogether of tobacco; only that steps should be taken to prevent the all too prevalent abuse of it. If such steps are now taken, the Army will owe Sir Thomas Fraser a larger debt than it will probably realise.

### ANTI-VENEREAL CRUSADE IN DERBYSHIRE.

THANKS to the enthusiasm and resourcefulness of Dr. S. Barwise, the chief medical officer for the county, and the far-sighted interest of its Public Health Committee, Derbyshire will rank as among the pioneers in the campaign against venereal diseases. The question came before the County Council last week, and instructions were given for the preparation of a comprehensive scheme for carrying into effect the new regulations for the prevention and treatment of the disease. Dr. Barwise reported that facilities could be provided at the county laboratory for aiding diagnosis, and it was decided to ask the Local Government Board to recognise the laboratory, not only for the county but for any other authorities, and to approve of the pathological work being undertaken by Dr. Sheila Ross, who has been appointed temporary bacteriologist in place of the late Dr. Thomson, at a salary of £400 a year. With regard to treatment, it is proposed, if possible, to enter into contracts with the Derbyshire Royal Infirmary, Chesterfield Hospital, Burton-on-Trent Infirmary, and Stockport Infirmary, and to start out-patient clinics at these institutions, and at the Chinley and Ilkeston Tuberculosis Dispensaries. The treatment of women will in all probability be undertaken by a medical woman.

### DEATH OF AN EX-HOSPITAL SURGEON.

To those who knew the late Mr. W. A. Meredith, the news of his fatal accident whilst still in the enjoyment of robust health and undiminished zest in life will come as a painful shock. Mr. Meredith, who was sixty-nine years of age, retired from London and from practice a few years ago, and is already hardly more than a memory to the younger generation of surgeons. But in the 'eighties and 'nineties he was one of the foremost of the band of surgeons who applied the doctrines of Lister to the surgery of the abdomen, then almost *terra incognita* from the surgical point of view. Although he held but one surgical appointment (at the Samaritan Hospital for Women), Meredith's influence spread widely in London surgical circles in those days; and his technique, which was largely original, found many followers. He was of French-Canadian ancestry, but by birth an American citizen, becoming later a naturalised Englishman. He graduated at Edinburgh in 1872, having qualified as M.R.C.S. the previous year; was a frequent contributor to surgical literature, especially to the "Transactions of the Clinical Society"; and was a Justice of the Peace for the county of Norfolk, in which he lived after his retirement. His amiable personality secured him many friends, by all of whom his death will be greatly regretted.

### DEATH OF A GALLANT MEDICAL OFFICER.

GALLANTRY amongst officers of the Royal Army Medical Corps is common enough, as we are all aware; but few have surpassed the record of Dr. T. L. Ingram, M.C., D.S.O., whose death on the battlefield while attending to the wounded under fire has just been announced. When a medical student he served as a trooper in the Middlesex Yeomanry in South Africa, and established a fine reputation among his comrades for cheerful and imperturbable bravery. Finishing his medical education, begun at Cambridge University, at the London Hospital after the Boer War, Dr. Ingram became house surgeon at the Westminster Hospital and, later on, settled down to country practice. Volunteering again for service in this war, he became medical officer to a battalion of the Shropshire Light Infantry, and speedily attracted notice by his repeated acts of devotion and courage. In due time, he was rewarded with the Military Cross, and, later on, the D.S.O.; and it was by continuing to face the greatest risks in getting the wounded collected that he met his death. Dr. Ingram was not a man of robust physique, but his pluck triumphed over all physical disabilities, and his record has been equalled by few even in the R.A.M.C.

### THE FRIENDLY SOCIETY DISPUTE IN NEW ZEALAND.

THE details of a dispute between the Wellington Branch (New Zealand) of the British Medical Association and the Friendly Societies of that city were given in THE HOSPITAL a short time ago (September 16, 1916, p. 570). A curious development of the situation has since occurred, which is at once a testimonial to the British Medical Association's solidity and power in Wellington and a warning to those who would defy the general standard of ethical conduct among their professional brethren. In our last notice of this quarrel it was stated that the Friendly Societies had refused to grant the increased contract rates demanded by the doctors, and had appointed four qualified men at guaranteed salaries of £700 and extras. One of these, a Dr. E. W. Smythe, found his position so intolerable that he promptly resigned, four days after taking up his work. The trustees of the Society, which had appointed him thereupon brought an action against him for the recovery of three months' salary in lieu of notice. It seems that his salary was fixed at £500, not £700, so the amount claimed was £125 plus costs. The action was undefended, and judgment was entered for the amount claimed. It was stated in evidence that the Society had, through the default of Dr. Smythe, to fall back upon the scale rates, and had in three months paid out £900 to the doctors. This significant admission shows that the Friendly Societies were trying to get £900 worth of work done for less than a seventh of that sum, a point which the acute democracy of New Zealand is not likely to miss.

### THE MEDICAL SOCIETY OF LONDON.

THE opening meeting of the Society was held on Monday, 9th inst., when the new President, Lieutenant-Colonel D'Arcy Power, F.R.C.S., delivered an address on "John Ward and his Diary." Ward, he said, has long been known as the Vicar of Stratford-on-Avon, who left on record, as a matter of current gossip, that "Shakespear, Drayton, and Ben Jonson had a merry meeting and itt seems drunk too hard for Shakespear died of a feavour there contracted." The passage occurs in one of the sixteen note-books which have long been in the possession of the Medical Society of London, from which Dr. Severn published extracts in 1839. Lieutenant-Colonel Power has now re-examined the earlier volumes, and gave an account of the results in his address. The volumes, he showed, are in no sense diaries, but rather commonplace books, in which were recorded the thoughts, actions, and interests of a writer who "sate at our table at Christ Church," Oxford, from 1652 to 1660. During this time nearly all the original members of the Royal Society were resident in Oxford. Ward was on terms of intimacy with many of them, and appears to have assisted in the work of several. His notes, therefore, are a faithful reflection of the interests of a resident graduate during one of the most fruitful periods of the University's existence. Dr. William Harvey, Warden of Merton, was working at Trinity with Dr. Bathurst on the incubation of the chick. Dr. Willis was in practice at the back of Christ Church and was directing the work of his two young assistants, "Dick" Lower and Christopher Wren. Dr. Wallis, the friend of Samuel Pepys, had not yet made his reputation as a mathematician and was working at the circulation of the blood. Barlow was studying Oriental languages at the Bodleian. Coming to the University as a gentleman of fortune and the son of a father who had suffered at Naseby, Ward's eager and enthusiastic nature soon assimilated the new spirit of inquiry, and the President gave numerous instances of the interest he displayed in every branch of science. Coming to London, he studied medicine for a year or two, but was unable to decide whether he should obtain a degree in medicine from a foreign University or enter the Church. Eventually he took Orders, and got himself presented to the vicarage of Stratford-on-Avon.

### PANEL DOCTORS AND CONSULTANTS.

DISCONTENT with their diminished remuneration and with other conditions of their work, such as surcharging, has been steadily simmering amongst the panel practitioners for some time. It seems possible that this chronic irritation and disgust may come to the boiling pitch on October 19, when a conference of Local Medical and Panel Committees will be held in London to discuss some new regulations issued by the Insurance Commissioners concerning the relations between panel practitioners and consultants. These regulations do, it is true, impose new duties on panellers without providing them with any extra remuneration; but they are not otherwise unreasonable, and if they do spur the panel men into rebellion, it will only be because they come on the top of repeated infringements of the panel contract in other respects.

### THIS WEEK'S DRUG MARKET.

THE downward tendency in the values of some of the synthetic drugs is less pronounced, and in one or two instances the tendency is rather steadier. For instance, aspirin, which had been slowly declining in price almost week by week for some little time, has become slightly dearer; this change in the tendency is probably temporary, and it would certainly seem to be unwise to purchase more than is necessary to satisfy immediate requirements. The price of salicylates is also rather steadier. Phenacetin and barbitone continue very scarce, and there seems to be little likelihood of any material decline in value during the next few weeks. A small quantity of resorcin is now available, but extremely high prices are wanted. The tendency in the value of bromides is distinctly firmer, and an advance in price would not come as a surprise. Both tartaric acid and citric acid tend downwards in price; makers of iron and quinine citrate have reduced their quotations. In consequence of increased buying of Persian opium the price has advanced. The advanced price of camphor is well maintained. Large stocks of ipecacuanha are available, and a reduction in price is not improbable. Liquid extract of male fern is scarce and dearer. The high price of sugar of milk is not quite so firmly maintained. Bergamot oil is cheaper. Lower prices are quoted for acetic acid. Chloral hydrate is slightly lower in value. Generally speaking, the tone of the drug market is steadier, and the general slump that had been expected in some quarters is slow to arrive, and is possibly a long way off.

